# Gliflozine – in Zukunft Kardioprotektiva?

**DOI:** 10.1007/s00108-021-01083-0

**Published:** 2021-06-23

**Authors:** Ursula Rauch-Kröhnert, Ulf Landmesser

**Affiliations:** Klinik für Kardiologie, Campus Benjamin Franklin, Charité – Universitätsmedizin Berlin, Hindenburgdamm 30, 12200 Berlin, Deutschland

**Keywords:** „Sodium-glucose-transporter-2“-Inhibitoren, Diabetes mellitus, Typ 2, Herzinsuffizienz, Antidiabetika, Medikamentenzulassung, Sodium-glucose transporter 2 inhibitors, Diabetes mellitus, type 2, Heart failure, Antidiabetics, Drug approval

## Abstract

Gliflozine (Inhibitoren der „sodium-dependent glucose cotransporter“, SGLT) sind Arzneistoffe, die ursprünglich zur Behandlung des Diabetes mellitus eingesetzt und der Gruppe der Antidiabetika zugeordnet wurden. Seit November 2020 ist mit Dapagliflozin erstmalig ein SGLT2-Inhibitor zur Behandlung von Patienten mit Herzinsuffizienz (mit reduzierter linksventrikulärer Funktion), unabhängig vom Diabetesstatus, zugelassen worden. Das Präparat Empagliflozin hat gerade – im Juni 2021 – von der europäischen Arzneimittelbehörde (EMA) eine Zulassung für die Therapie der Herzinsuffizienz mit reduzierter Ejektionsfraktion erhalten. Somit stehen verschiedene Gliflozine nicht nur zur Behandlung des Diabetes mellitus, sondern auch der Herzinsuffizienz zur Verfügung. Der vorliegende Beitrag vermittelt Grundlagenkenntnisse zu den Gliflozinen und bietet eine Übersicht zur ihrer Bedeutung sowohl in der Behandlung des Diabetes mellitus Typ 2 als auch aufgrund ihrer kardio- und nephroprotektiven Funktion.

## Abstract (en)

Gliflozine (inhibitors of sodium-dependent glucose cotransporters, SGLT) are medications which were originally used in the treatment of diabetes mellitus and are assigned to the group of antidiabetics. Since November 2020 the SGLT2 inhibitor dapagliflozin has been approved for the treatment of heart failure (with reduced left ventricular function) for the first time, independent of the diabetes status. The substance empagliflozin has just received an approval for the treatment of heart failure with reduced ejection fraction from the European Medicines Agency (EMA). Therefore, different gliflozins are now available not only for the treatment of diabetes mellitus but also for the treatment of cardiac insufficiency. This article mediates fundamental knowledge on the gliflozins and provides an overview of the importance in the treatment of type 2 diabetes mellitus as well as cardioprotective and nephroprotective functions.

## Gliflozine

Gliflozine (Inhibitoren der „sodium-dependent glucose cotransporter“, SGLT) sind Arzneistoffe, die ursprünglich zur Behandlung des Diabetes mellitus eingesetzt und der Gruppe der Antidiabetika zugeordnet wurden. Seit November 2020 ist mit Dapagliflozin erstmalig ein SGLT2-Inhibitor zur Behandlung von Patienten mit Herzinsuffizienz (mit reduzierter linksventrikulärer [LV-]Funktion, HFrEF), unabhängig vom Diabetesstatus, zugelassen worden. Das Präparat Empagliflozin hat gerade – im Juni 2021 – von der europäischen Arzneimittelbehörde (EMA) eine Zulassung für die Therapie der Herzinsuffizienz mit reduzierter Ejektionsfraktion erhalten. Somit stehen verschiedene Gliflozine nicht nur zur Therapie des Diabetes mellitus, sondern auch der Herzinsuffizienz zur Verfügung.

### Präparate

In Deutschland sind derzeit 4 Gliflozine zugelassen, die als Monopräparate oder in Kombination mit anderen oralen Antidiabetika verfügbar sind.

- Dapagliflozin (Forxiga®, in Xigduo®),
- Empagliflozin (Jardiance®, in Glyxambi®),
- Ertugliflozin (in Steglujan®),
- Sotagliflozin (Zynquista®, nicht auf dem Markt verfügbar).

Der Vertrieb von Canagliflozin (Monopräparat Invokana® und Kombipräparat Invokamet®) wurde in Deutschland 2014 eingestellt, da diese Präparate nach einer für den Hersteller enttäuschenden Nutzenbewertung vom Markt genommen wurden.

Folgende Gliflozine wurden bisher nicht in Deutschland zugelassen:

- Ipragliflozin,
- Remogliflozin,
- Sergliflozin,
- Tofogliflozin.

### Wirkmechanismus

Gliflozine verbessern die Blutzuckerkontrolle durch die Hemmung des natriumabhängigen Glucosetransporter 2 („sodium-dependent glucose cotransporter 2“, SGLT2). Das SGLT2-Protein im proximalen Tubulus der Nieren ist für ca. 90 % der Glucoserückresorption verantwortlich, folglich kommt es durch die SGLT2-Hemmung zur vermehrten Glucoseausscheidung über den Harn (Glucosurie). Dapa‑, Empa- und Ertugliflozin sind SGLT2-spezifische Inhibitoren [1, 2].

Die SGLT2-Hemmer weisen chemische Ähnlichkeit mit dem Wirkstoff Phlorizin, einem O‑Glykosid, auf, der schon 1835 aus Apfelbaumrinde isoliert, sich durch unzureichende Bioverfügbarkeit nicht für die Diabetesbehandlung eignete. Sie wirken unabhängig vom Insulinstoffwechsel. Bei kompletter Hemmung von SGLT2 kann der Patient 50–100 g Glucose/Tag über den Harn verlieren; dies entspricht fast 30 % der täglichen Kohlenhydrataufnahme und führt zu der beobachtbaren Gewichtsabnahme [3]. Zusammen mit der Glucose- wird die Natriumausscheidung erhöht; dies bedingt eine osmotische Diurese, wodurch es zur Verminderung des intravaskulären Volumens und zur leichten Absenkung des Blutdrucks kommt [3]. Die durch Gliflozine verursachte Glucoseausscheidung nimmt bei niedrigen Blutzuckerspiegeln ab [4], sodass unter der Gliflozingabe ein nur sehr geringes Hypoglykämierisiko zu verzeichnen ist.

Gliflozine erhöhen sowohl die Glucose- als auch die Natriumausscheidung

Gliflozine scheinen auch den sarkolemmalen Na^+^/H^+^-Exchanger am Herzen zu hemmen, wodurch die intrazelluläre Natriumkonzentration im Myokardgewebe reduziert wird. Eine Verminderung des bei Herzinsuffizienz bekannterweise in Kardiomyozyten erhöhten intrazellulären Natriumgehalts könnte die unter der Gliflozintherapie beobachtete Verbesserung der Herzinsuffizienz erklären. Da erhöhte intrazelluläre Natriumkonzentrationen das mitochondriale Ca^2+^-Handling beeinflussen, verschlechtert sich die Energieversorgung des Kardiomyozyten im Rahmen der Herzinsuffizienz. Somit trägt die Behandlung mit einem Gliflozin bei Vorliegen einer Herzinsuffizienz möglicherweise durch Verbesserung der mitochondrialen Funktion zum Erhalt der myokardialen Pumpfunktion bei.

### Wirkung

**Figure 1:**
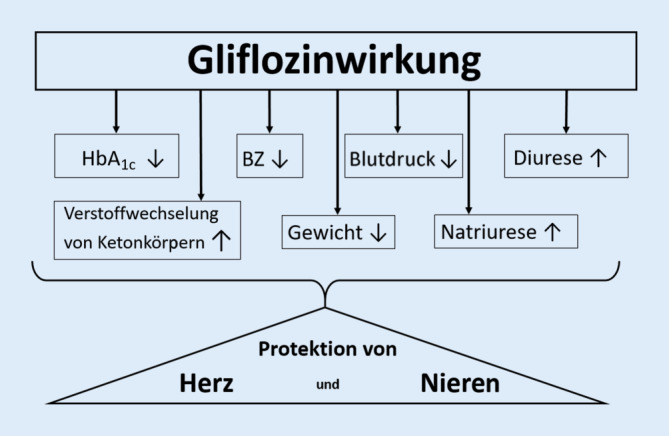

Die SGLT2-Inhibitoren können die Blutglucosewerte durchschnittlich um bis zu 30 mg/dl und den HbA_1c_-Wert durchschnittlich um ca. 0,6 % senken. Weiterhin bewirken SGLT2-Hemmer eine leichte Gewichtsabnahme (ca. 1,3–2,0 kg) und eine mäßige Blutdrucksenkung. Bedeutsam sind die in den klinischen Studien beobachteten kardio- und nephroprotektiven Wirkungen der SGLT2-Hemmung (Abb. 1).

### Indikationen

- Diabetes mellitus Typ 2 (alle Gliflozine),
- chronische Herzinsuffizienz mit reduzierter Pumpfunktion (Dapagliflozin und Empagliflozin),
- Diabetes mellitus Typ 1 (nur Dapagliflozin und Sotagliflozin; die Indikationsstellung beim Typ-1-Diabetes sollte eher Spezialisten vorbehalten bleiben).

### Mögliche Nebenwirkungen

Die SGLT2-Inhibitoren weisen u. a. folgende Nebenwirkungen auf:

- Harnwegsinfektionen bzw. genitale Infektionen (v. a. vulvovaginale Kandidosen, Balanitis, Balanoposthitis), wahrscheinlich bedingt durch ein beschleunigtes Wachstum von Mikroorganismen im vermehrt Glucose enthaltenden Urin,
- Hypoglykämien,
- Polyurie mit Dehydratation, nachfolgend Schwindel bzw. Hypotonie,
- Leberfunktionsstörungen,
- allergische Reaktionen,
- nekrotisierende Fasziitis des Perineums (s. unten),
- diabetische Ketoacidose (s. unten).

#### Nekrotisierende Fasziitis des Perineums (Fournier-Gangrän)

In der Allgemeinbevölkerung beträgt die Inzidenz der Fournier-Gangrän 1,6/100.000 Männer und Jahr; die Erkrankung ist damit sehr selten, bei Frauen noch deutlich seltener. Einige wenige Fälle nach Markteinführung der SGLT2-Inhibitoren standen im Verdacht, in Verbindung mit dem Auftreten der Fournier-Gangrän zu stehen, und führten 2019 zur Verfassung eines Rote-Hand-Briefs.

#### Gliflozinassoziierte atypische diabetische Ketoacidose

Bisherige Untersuchungen ergaben, dass unter einer Behandlung mit Gliflozinen zwischen 12 und 20 % der Typ-2-Diabetes-Patienten asymptomatische Erhöhungen des Ketonkörpers β‑Hydroxybutyrat aufweisen [5–7]. Zu den diagnostischen Kriterien einer diabetischen Ketoacidose gehören neben dem Nachweis von Ketonkörpern ein pH-Wert ≤ 7,3, eine Serum-Bikarbonat-Konzentration ≤ 15 mmol/l und ein Anionen-Gap > 12 mmol/l [8]. Eine Besonderheit der gliflozinassoziierten diabetischen Ketoacidosen stellen – bedingt durch die vermehrte Glucoseausscheidung über die Nieren – die nur mäßig erhöhten bzw. teilweise sogar normwertigen Blutglucosekonzentrationen dar. Risikofaktoren für diabetische Ketoacidosen sind u. a. größere chirurgische Eingriffe, Infektionen, diätetische Nahrungsumstellungen oder Dosisreduktionen von Insulin (Tab. 1).

**Table 1:** 

Risikosituation	Maßnahmen in Bezug auf Gliflozine
Akute Erkrankung (z. B. Infektion, Gastroenteritis, Myokardinfarkt, Schlaganfall)	Pausierung Nach Besserung des klinischen Zustands sowie Normalisierung der Flüssigkeits- und Nahrungsaufnahme Einnahme fortsetzen
Bariatrische Chirurgie	Pausierung bei präoperativer Diät Indikation für Gliflozin postoperativ reevaluieren
Größere operative Eingriffe	Drei Tage vor dem Eingriff pausieren Nach Besserung des klinischen Zustands sowie Normalisierung der Flüssigkeits- und Nahrungsaufnahme Einnahme fortsetzen
Drohende Dehydratation (z. B. Vorbereitung zur Koloskopie, exzessiver Sport)	Pausierung, bis die Dehydratation behoben ist
Low-Carb-Diät	Pausierung bis zur Wiederaufnahme der normalen Ernährung
Exzessiver Alkoholkonsum	Sofortige Pausierung Indikation für Gliflozin zu späterem Zeitpunkt reevaluieren

Insgesamt sind gliflozinassoziierte Ketoacidosen sehr selten [9–11]. In Anbetracht der hohen Morbidität ist es entscheidend, diese durch Messung der Ketonkörper im Blut frühzeitig zu diagnostizieren, um eine entsprechende Therapie einleiten zu können.

### Einzelne Präparate und Auswahl ihrer wichtigsten klinischen Studien

#### Empagliflozin

##### Wichtige Studie.

Die EMPA-REG-OUTCOME-Studie war die erste große prospektive, randomisierte klinische Phase-III-Studie, die die protektive kardiovaskuläre Wirkung, die mit einer Empagliflozinbehandlung assoziiert ist, bei Patienten mit Diabetes mellitus Typ 2 für eine Dauer bis zu 4,5 Jahren (mediane Behandlungsdauer 3,1 Jahre) dokumentierte. In dieser Studie wurde an 7020 Patienten mit koronarer Herzkrankheit (Ein- und Mehrgefäßerkrankung), Zustand nach Myokardinfarkt (MI), Zustand nach ischämischem Schlaganfall und/oder peripherer arterieller Verschlusskrankheit untersucht, inwiefern eine Therapie mit Empagliflozin im Vergleich zu Placebo das Risiko des Auftretens von kardiovaskulären Ereignissen bei Patienten mit Typ‑2 Diabetes mellitus und kardiovaskulären Vorerkrankungen beeinflusst. Zusätzlich zu der vorbestehenden Therapie erhielten die Patienten entweder 10 mg Empagliflozin (*n* = 2345), 25 mg Empagliflozin (*n* = 2342) oder Placebo (*n* = 2333) [12].

Das Durchschnittsalter der Patienten betrug 63 Jahre, es waren 9,3 % der Patienten ≥ 75 Jahre alt, und 71,5 % der Teilnehmer waren männlich.

Die EMPA-REG-OUTCOME-Studie war zur Bewertung des kardiovaskulären Risikos unter der Therapie mit Empagliflozin konzipiert. Nach der Anfangsperiode von 12 Wochen konnte die blutzuckersenkende Behandlung gemäß den aktuellen Therapierichtlinien angepasst werden. Weitere Begleitmedikationen wurden während der gesamten Studie optimiert.

Als primärer Endpunkt wurde die Zeit bis zum ersten Ereignis des kombinierten Endpunkts aus kardiovaskulären Todesfällen, nichttödlichen MI und nichttödlichen Schlaganfällen, der „3-point MACE“, erfasst (MACE: „major adverse cardiovascular events“).

##### Reduktion des Risikos für kardiovaskuläre Todesfälle und Gesamtmortalität.

Empagliflozin war im Vergleich zur Placebobehandlung hinsichtlich des primären Endpunkts überlegen (10,5 % vs. 12,1 %; „hazard ratio“ [HR] 0,86; 95 %-Konfidenzintervall [95 %-KI] 0,74–0,99, *p* für Überlegenheit = 0,0382) und führte zu einer signifikanten Senkung des Auftretens kardiovaskulären Ereignisse. Die signifikante Reduktion des primären Endpunkts beruhte wesentlichen auf der Senkung der Inzidenz kardiovaskulärer Todesfälle (Empagliflozin vs. Placebo, 3,7 % vs. 5,9 % [HR 0,62; 95 %-KI 0,49–0,77, *p* < 0,001]). Die Reduktionen der MACE-Rate sowie der Häufigkeit stationärer Aufnahmen aufgrund von Herzinsuffizienz traten unabhängig von der HbA_1c_-Senkung auf [13]. Weder das Risiko des nichttödlichen MI noch die Rate der Schlaganfälle wurde durch die Behandlung mit Empagliflozin im Vergleich zur Placebobehandlung signifikant gesenkt. Resultierend aus der Verringerung kardiovaskulärer Todesfälle sank unter der Therapie mit Empagliflozin auch die Gesamtmortalität (5,7 % vs. 8,3 %; HR 0,84; 95 %-KI 0,60–1,16; *p* < 0,001).

##### Reduktion renaler Ereignisse.

In einer separaten explorativen Auswertung der EMPARE-REG-OUTCOME-Studie wurden der Einfluss von Empagliflozin auf die diabetische Nephropathie und die Inzidenz von renalen Ereignissen analysiert [14]. Kriterien für das erstmalige Auftreten und die Verschlechterung der Nephropathie waren die Entwicklung einer Makroalbuminurie, eine Verdopplung der Serum-Kreatinin-Konzentration, der Beginn einer Nierenersatztherapie und renal verursachter Tod. Das Risiko für diesen renalen Endpunkt wurde durch Empagliflozin signifikant relativ um 39 % (*p* < 0,001) im Vergleich zu Placebo verringert.

Welchen Einfluss Empagliflozin speziell auf die diabetische Nephropathie und die Inzidenz von renalen Ereignissen als sekundäre Endpunkte hatte, zeigte eine explorative Analyse an rund einem Viertel der Studienteilnehmer mit renaler Funktionseinschränkung. Bei 17,8 % der Studienteilnehmer betrug die geschätzte glomeruläre Filtrationsrate (eGRF) im Bereich zwischen 45 und 59 ml/min und bei 7,7 % im Bereich zwischen 30 und 44 ml/min. Bei 28,7 % der Studienteilnehmer bestand zu Beginn eine Mikroalbuminurie und bei 11,0 % bereits eine Makroalbuminurie.

Das Risiko für eine Progression zur Makroalbuminurie wurde durch Empagliflozin relativ um 38 % signifikant verringert (Inzidenz: 11,2 % vs. 16,2 %), und das Risiko für eine Verdopplung der Serum-Kreatinin-Konzentration um 44 % (1,5 % vs. 2,6 %). Das Risiko für die Einleitung einer Nierenersatztherapie war nur etwa halb so hoch wie unter Placeboanwendung (0,3 % vs. 0,6 %, Risikoreduktion: 55 %). Anhand serieller eGRF-Messungen konnte gezeigt werden, dass Empagliflozin die Nierenfunktion – nach einem kurzfristigen initialen eGRF-Abfall – über längere Zeit stabilisierte, während unter Placebo eine stetige Abnahme zu verzeichnen war.

Ferner waren Körpergewicht und Blutdruckwerte von Patienten in der Empagliflozingruppe durchschnittlich niedriger als in der Placebogruppe. Die beobachteten Unterschiede sind allerdings zu gering, um die günstigen renalen Effekte von Empagliflozin hinreichend erklären zu können. Es wird derzeit von einem direkten Schutzmechanismus ausgegangen, den das Gliflozin auf die Nierenfunktion ausübt.

#### Dapagliflozin

##### Wichtige Studie.

Eine weitere Phase-3-Studie, DECLARE-TIMI 58, untersuchte den kardiovaskulären Benefit von Dapagliflozin bei Patienten mit Typ-2-Diabetes. Die DECLARE-TIMI-58-Studie wurde weltweit in 33 Ländern durchgeführt. Insgesamt nahmen 17.160 Patienten mit Typ-2-Diabetes teil, bei denen aufgrund manifester Herz-Kreislauf-Erkrankungen oder lediglich aufgrund multipler Faktoren – und dazu gehörte auch schon das Alter – ein erhöhtes kardiovaskuläres Risiko bestand. Die Patienten wurden über einen Zeitraum von im Median 4,2 Jahren beobachtet. Es erfolgte eine Therapie mit Dapagliflozin oder die Gabe von Placebo; beide wurden als Add-on zum üblichen „standard of care“ verabreicht [15].

Wie bei den anderen kardiovaskulären Wirksamkeitsstudien wurde der primäre, zusammengesetzte Wirksamkeitsendpunkt aus Hospitalisierung aufgrund von Herzinsuffizienz oder kardiovaskulärem Tod (4,9 % vs. 5,8 %; HR 0,83; 95 %-KI 0,73–0,95, *p* = 0,005 für Überlegenheit) signifikant erreicht, was einer relativen Risikoreduktion von 17 % entsprach. Dieser Endpunkt wurde v. a. durch eine Reduktion von Hospitalisierungen aufgrund einer Herzinsuffizienz von 27 % (HR 0,73; 95 %-KI 0,61–0,88, *p* < 0,001) getrieben, während die Rate kardiovaskulärer Todesfälle sich nicht signifikant unterschied (HR 0,98; 95 %-KI 0,82–1,17). Im Unterschied zu den anderen beiden großen kardiovaskulären Wirksamkeitsstudien ergab sich in dieser Untersuchung an deutlich weniger kranken Patienten allerdings keine signifikante Senkung atherosklerotischer Ereignisse. Die MACE-Rate betrug in der Dapagliflozingruppe 8,8 % und in der Placebogruppe 9,4 % (HR 0,93; 95 %-KI 0,84–1,03, *p* = 0,17 für Überlegenheit). Da der Großteil der Patienten, die in die Studie DECLARE-TIMI-58 aufgenommen wurden, allein aufgrund bestehender Risikofaktoren randomisiert wurde, unterscheidet sich diese Studie bezüglich der Charakteristika der eingeschlossenen Patienten von den Studien EMPA-REG-OUTCOME und CANVAS.

Ein klarer Nutzen zeigte sich mit Blick auf die Nierenfunktion. Dapagliflozin reduzierte das Risiko des kombinierten renalen Endpunkts, bestehend aus einem eGRF-Abfall um mindestens 40 % auf weniger als 60 ml/min und 1,73m^2^KOF, terminaler Niereninsuffizienz und renal oder kardiovaskulär bedingtem Tod, um 24 % (4,3 % vs. 5,6 %; HR 0,76; 95 %-KI 0,67–0,87, *p* < 0,01). Diese Befunde stehen im Einklang mit denen der anderen beiden kardiovaskulären Endpunktstudien.

##### Metaanalysen zu den 3 großen kardiovaskulären Endpunktstudien EMPA-REG-OUTCOME, DECLARE-TIMI 58 und CANVAS.

Die deutliche Risikoreduktion und Vermeidung schwerwiegender Folgekomplikationen des Diabetes belegen die 3 großen kardiovaskulären Outcome-Studien zu den SGLT2-Inhibitoren EMPA-REG-OUTCOME, CANVAS und DECLARE-TIMI 58.

Typ-2-Diabetes-Patienten profitieren bezüglich der Herzinsuffizienz von der SGLT2-Hemmer-Behandlung

Mit Blick auf das Fortschreiten der Herzinsuffizienz zeigte die Metaanalyse dieser Studien [16], dass Typ-2-Diabetes-Patienten bezüglich der Herzinsuffizienz eindeutig von einer Behandlung mit SGLT2-Hemmern profitieren (s. unten). Dieser Effekt war unabhängig davon, ob zu Therapiebeginn bereits eine Herzinsuffizienz bestand oder nicht. Das Risiko, eine fortschreitende Niereninsuffizienz zu entwickeln, war laut der Metaanalyse unter Anwendung von SGLT2-Hemmern ebenfalls signifikant reduziert (s. unten). Die Metaanalyse spricht dafür, bei Patienten mit sich abzeichnender Nephropathie frühzeitig eine Behandlung mit SGLT2-Hemmern einzuleiten.

Ferner ergab sie bei Patienten mit Typ‑2 Diabetes und mindestens 2 kardiovaskulären Risikofaktoren, aber ohne atherosklerotische Gefäßerkrankung, keine signifikante Reduktion der Inzidenz von MI, Schlaganfällen und kardiovaskulären Todesfällen unter der Therapie mit einem Gliflozin. Hingegen sank bei Patienten mit manifester atherosklerotischer Erkrankung das Risiko kardiovaskulärer Ereignisse unter der Behandlung mit einem Gliflozin um 11 % (HR 0,89; *p* = 0,0014) ab.

#### Ertugliflozin

Die Kombination aus Ertugliflozin und Sitagliptin stellt die einzige Fixkombination eines SGLT2-Inhibitors mit einem Dipeptidylpeptidase-4(DPP-4)-Hemmer dar und ist bei Erwachsenen mit Typ-2-Diabetes mellitus ab 18 Jahren zusätzlich zu Diät und Bewegung zur Verbesserung der Blutzuckerkontrolle indiziert. Das Kombinationspräparat kann sowohl bei Patienten, deren Blutzuckerkonzentration unter Metformin und/oder einem Sulfonylharnstoff und Sitagliptin oder Ertugliflozin nicht ausreichend gesenkt wurde, eingesetzt werden.

Die kardiovaskuläre Sicherheit von Sitagliptin bei Patienten mit Typ-2-Diabetes wurde in der TECOS-Studie nachgewiesen [17]. Das Risiko der Hypoglykämie war in der Studie gering; das Gewicht der Patienten blieb konstant.

Basis für die Zulassung des Kombinationspräparates aus Ertugliflozin und Sitagliptin waren die Ergebnisse des klinischen Entwicklungsprogramms E*v*aluation of *Ert*ugliflozin eff*i*cacy and *s*afety (VERTIS), das 9 Phase-III-Studien mit rund 12.600 an Typ-2-Diabetes erkrankten Erwachsenen beinhaltete. Dabei wurde das Konzept der oralen Dreifachkombination in der VERTIS-SITA2-Studie untersucht [18]. In VERTIS SITA2, einer 52-wöchigen, doppelblinden, prospektiven Studie wurde Ertugliflozin im Vergleich zu Placebo Patienten mit Typ-2-Diabetes, deren Blutzuckerspiegel unter Anwendung von Metformin (≥ 1500 mg/Tag) und Sitagliptin (100 mg/Tag) bei einem HbA_1c_-Ausgangswert von 7,0–10,5 % nicht ausreichend kontrolliert werden konnte, verabreicht. Zusätzlich zu Metformin und Sitagliptin erhielten insgesamt 621 in die Studie eingeschlossenen Patienten in randomisierter Weise entweder 5 mg Ertugliflozin/Tag oder 15 mg Ertugliflozin/Tag oder Placebo. Nach 26 Wochen hatte die zusätzliche Gabe von 5 mg Ertugliflozin/Tag zu einer signifikanten Senkung des HbA_1c_-Werts von 0,8 % bzw. die Gabe von 15 mg Ertugliflozin/Tag zu einer signifikanten HbA_1c_-Wert-Senkung von 0,9 % geführt, im Vergleich zu 0,1 % unter Placebo (jeweils *p* < 0,001 vs. Placebo).

Die Gabe von 5 mg bzw. 15 mg Ertugliflozin/Tag führte zu einer Reduktion des Körpergewichtes um 3,3 kg bzw. 3 kg im Vergleich zu 1,3 kg unter Placebo. Der systolische Blutdruckwert wurde im Vergleich zu Placebo ebenfalls signifikant um 3,8 mm Hg unter der Gabe von 5 mg Ertugliflozin/Tag und um 4,8 mm Hg unter der Gabe von 15 mg Ertugliflozin/Tag vs. 0,9 mm Hg unter Placeboanwendung gesenkt.

Die orale Dreifachtherapie mit Metformin plus DPP-4-Inhibitor plus Ertugliflozin erwies sich als wirksam und sicher.

#### Sotagliflozin

Sotagliflozin hemmt im Unterschied zu anderen Gliflozinen auch den natriumabhängigen Glucosekotransporter SGLT1, der an der Glucoseaufnahme im Gastrointestinaltrakt beteiligt ist. Der duale Wirkmechanismus von Sotagliflozin bietet wichtige Behandlungsvorteile für Erwachsene mit Typ-1-Diabetes. Im Jahr 2017 wurde Sotagliflozin im Rahmen der klinischen Studie InTandem‑3 erstmals bei 3000 Patienten mit Diabetes mellitus Typ 1 auf Sicherheit und Wirksamkeit in Kombination mit einer Insulintherapie untersucht [19]. Bei guter Verträglichkeit zeigten sich positive Wirkungen auf den HbA_1c_-Wert sowie auch auf das Körpergewicht und den systolischen Blutdruck.

In der randomisierten doppelblinden SOLOIST-WHF-Studie fand bei 1222 Diabetespatienten, die wegen dekompensierter Herzinsuffizienz stationär mithilfe einer i.v.-Diuretika-Therapie rekompensiert wurden, eine Behandlung mit Sotagliflozin (400 mg/Tag, *n* = 608) oder Placebo (*n* = 614) statt. Es wiesen 80 % aller Teilnehmer eine linksventrikuläre Ejektionsfraktion < 50 % auf. Die Nachbeobachtungszeit war mit 9 Monaten aufgrund der Coronapandemie kürzer als geplant. Sotagliflozin reduzierte die Inzidenz des kardiovaskulären Todes oder der Klinikeinweisungen aufgrund von Herzinsuffizienz im Vergleich zur Placebogruppe auch bei diesen erst kürzlich dekompensierten Patienten um 33 % (relative Risikoreduktion [HR] 0,67; 95 %-KI 0,52–0,85, *p* < 0,001). Ferner war diese Wirkung unabhängig von der linksventrikulären Ejektionsfraktion auch bei den ca. 20 % der in die Studie eingeschlossenen Patienten mit einem „heart failure with preserved ejection fraction“ (HFpEF) nachweisbar [20].

Die multizentrische doppelblinde Studie SCORE untersuchte, ob Sotagliflozin eine Prävention kardiovaskulärer Ereignisse bei Patienten mit Diabetes (HbA_1c_ ≥ 7 %) und chronischer Niereninsuffizienz (mit und ohne Albuminurie, geschätzte GFR 25–60 ml/min und 1,73 m^2^KOF) bewirkt [21]. Die Patienten (*n* = 10.584) wurden entweder mit Sotagliflozin oder Placebo behandelt und im Mittel 16 Monate nachbeobachtet. Sotagliflozin reduzierte die Ereignisrate aus kardiovaskulär bedingtem Tod oder Hospitalisierungen sowie Notfallbehandlungen wegen Herzinsuffizienz auf 5,6/100 Patientenjahre vs. 7,5/100 Patientenjahre in der Placebogruppe (HR 0,74, 95 %-KI 0,63–0,88, *p* < 0,001). Für den ursprünglichen koprimären Endpunkt, das Auftreten eines kardiovaskulären Todes, eines nichttödlichen MI sowie eines nichttödlichen Schlaganfalls, ergab sich eine HR von 0,84 (95 %-KI 0,72–0,99). Allerdings wurden unter der Sotagliflozinbehandlung auch mehr Nebenwirkungen berichtet, besonders Diarrhöen und genitale Pilzinfektionen. Aufgrund der Einstellung der Finanzierung wurde die Studie SCORE vorzeitig beendet. Sotagliflozin ist derzeit nicht auf dem deutschen Markt verfügbar.

#### Fazit – starke Empfehlung für den Einsatz von Gliflozinen bei Patienten mit Diabetes mellitus Typ 2

Inzwischen wird der frühe Einsatz von SGLT2-Inhibitoren bei Patienten mit Diabetes mellitus Typ 2 mit hohem kardialen oder renalen Risiko von der American Diabetes Association (ADA), der European Association for the Study of Diabetes (EASD) und dem American College of Cardiology (ACC) empfohlen. Die European Society of Cardiology (ESC) empfiehlt in der aktuellen Leitlinie (2019) sogar erstmals den Einsatz von SGLT2-Inhibitoren bei Diabetespatienten mit hohem kardiovaskulärem Risiko vor einer Metformintherapie [22].

## Bedeutung für die Behandlung der chronischen Herzinsuffizienz

### Wichtige Studien

Aus den wichtigen Erkenntnissen zur positiven Wirksamkeit von Gliflozinen auf die Herzinsuffizienz bei Patienten mit Typ-2-Diabetes mellitus, den kardiovaskulären Sicherheitsstudien EMPA-REG-OUTCOME, CANVAS und DECLARE-TIMI 58, ergab sich die Fragestellung nach einer Wirksamkeit dieser Substanzen auf die Herzinsuffizienz, unabhängig vom Diabetesstatus. Diese wurde kürzlich in 2 großen randomisierten prospektiven Herzinsuffizienzstudien für Dapagliflozin und Empagliflozin an Patienten mit HFrEF nachgewiesen. Dabei wurden die jeweiligen Gliflozine zusätzlich zu einer leitliniengemäßen medikamentösen Herzinsuffizienztherapie eingesetzt. In beiden Studien wurde eine Reduktion des primären kombinierten Endpunktes, der Hospitalisierung aufgrund von Herzinsuffizienz oder des kardiovaskulären Todes, erreicht.

#### Die DAPA-HF-Studie.

In der DAPA-HF-Studie wurde mit Dapagliflozin erstmals ein SGLT2-Hemmer im Vergleich zu einer Placebobehandlung bei Patienten mit Herzinsuffizienz (Stadium ≥II der NYHA-Klassifikation) und reduzierter LV-Funktion auf dem Boden einer leitliniengerechten Herzinsuffizienztherapie unabhängig vom Diabetesstatus getestet [23]. Nach eineinhalb Jahren zeigten sich signifikante Reduktionen der als primäre Endpunkte definierten Parameter kardiovaskulärer Tod, herzinsuffizienzbedingte Hospitalisierung oder Notaufnahmekontakte wegen Herzinsuffizienz (16,3 % in der Dapagliflozingruppe vs. 21,2 % in der Placebogruppe; *p* < 0,001) um 26 %. Sowohl die kardiovaskuläre Mortalität (9,6 % in der Dapagliflozingruppe vs. 11,5 % in der Placebogruppe; *p* < 0,029) als auch die Gesamtmortalität (11,6 % in der Dapagliflozingruppe vs. 11,6 % in der Placebogruppe; *p* < 0,022) wurden in dieser Studie durch Dapagliflozin signifikant gesenkt. Der positive Effekt des Dapagliflozins auf die Herzinsuffizienz in der DAPA-HF-Studie war über alle Subgruppen konsistent und unabhängig von der Stoffwechseleinstellung. Die Auswertung der primären Endpunkte nach Behandlungstagen zeigte einen signifikanten Effekt in der Therapie der Herzinsuffizienz bereits nach 28 Tagen [24].

#### Die EMPEROR-reduced-Studie.

Nach der DAPA-HF wurde 2020 beim Kongress des European Society of Cardiology (ESC) die EMPEROR-reduced-Studie zur Herzinsuffizienz vorgestellt [25]. Als primärer Wirksamkeitsendpunkt wurde das Auftreten von kardiovaskulär verursachten Todesfällen und Klinikaufenthalten wegen einer Herzinsuffizienz erfasst. Eine Behandlung mit Empagliflozin, 10 mg/Tag, führte in einem medianen Behandlungszeitraum von 16 Monaten zu einer signifikanten Reduktion des primäreren Endpunkts im Vergleich zur Placebobehandlung (19,4 % in der Empagliflozingruppe vs. 24,7 % in der Placebogruppe; *p* < 0,001) mit einer relativen Risikoreduktion von 25 % im Vergleich zu Placebo. Maßgeblich für diese Risikoreduktion war die relative Reduktion von Hospitalisierungen aufgrund von Herzinsuffizienz um 31 % (HR 0,69; 95 %-KI 0,59–0,81; *p* < 0,001). Die kardiovaskuläre Mortalität betrug 10 % in der Empagliflozin- und 10,8 % in der Placebogruppe (HR 0,92; 95 %-KI 0,75–1,12) und war – wie die Senkung der Gesamtsterblichkeit – nicht signifikant.

Dapa- und Empagliflozin können bei Patienten mit HFrEF als Add-on etablierte Therapien ergänzen

Subgruppenanalysen ergaben, dass der klinische Benefit von Empagliflozin unabhängig von einer Kombinationstherapie mit Sacubitril und Valsartan auftrat. Der Therapierfolg war ebenfalls unabhängig vom Diabetesstatus. Somit erweisen sich die Gliflozine Dapa- und Empagliflozin als geeignete Therapeutika, die bei Patienten mit reduzierter LV-Funktion als Add-on bereits im klinischen Alltag etablierte Therapien der Herzinsuffizienz ergänzen können.

### Metaanalysen zur Herzinsuffizienz

Obwohl es in den beiden Studien Unterschiede im Hinblick auf die Senkung der kardiovaskulären Mortalität und Gesamtsterblichkeit und die renalen Endpunkte gab, zeigt die prädefinierte Metaanalyse der beiden großen Endpunktstudien, dass die Therapie mit einem SGLT2-Hemmer einen signifikant positiven Effekt auf die Verbesserung der Herzinsuffizienz und einen protektiven Effekt auf die Nierenfunktion bei Patienten mit einem „heart failure with reduced ejection fraction“ (HFrEF) hat [26, 27]. Man kann hier sicherlich von einem Klasseneffekt sprechen.

### Gabe von Gliflozinen bei Herzinsuffizienz trotz niedriger eGFR

Im Rahmen der Neueinstellung eines Diabetes sollte ein SGLT2-Hemmer nur ab einer eGFR von 60 ml/min und 1,73m^2^KOF eingesetzt werden. Die Glucoseausscheidung und damit auch der positive Einfluss auf den Stoffwechsel sind von der eGFR abhängig, deshalb müssen Gliflozingaben zur Behandlung des Diabetes aktuell bei einer eGFR unter 45 ml/min und 1,73m^2^KOF abgesetzt werden. Für die Therapie der Herzinsuffizienz werden andere Grenzwerte, wie sie in den beiden HF-Studien untersucht wurden, empfohlen: Für die Dapa-HF-Studie wurden Patienten mit einer eGFR ≥30 ml/min und 1,73m^2^KOF und für die EMPEROR-reduced-Studie mit einer eGFR ≥20 ml/min und 1,73m^2^KOF rekrutiert.

### Fazit – überzeugende Daten für den Einsatz von Dapa- und Empagliflozin bei HFrEF

In Anbetracht der Eindeutigkeit der Studienlage, die die hohe Wirksamkeit der Gliflozine bei Patienten mit HFrEF belegt, wurde jüngst Empagliflozin die Zulassung von der EMA für dieses Indikationsgebiet erteilt. Außerdem wurde Dapagliflozin als erster Vertreter der SGLT2-Inhibitoren vom G-BA (Gemeinsamer Bundesausschuss) ein beträchtlicher Zusatznutzen über alle Patientengruppen der DAPA-HF-Studie bescheinigt.

### Derzeit noch fehlende Studiendaten für den Einsatz der Gliflozine bei HFpEF

Während beim HFrEF therapeutisch wirksame Medikamente verfügbar sind, fehlen spezifische prognostisch-wirksame Therapieoptionen für Patienten mit erhaltener Ejektionsfraktion (HFpEF). Die Daten aus der SOLOIST-WHF-Studie zeigen, dass die positive Wirkung von Sotagliflozin auf den Progress der Herzinsuffizienz unabhängig von der LV-Ejektionsfraktion auch bei den in die Studie eingeschlossenen HFpEF-Patienten nachzuweisen war [20]. Diese Daten lassen hoffen, dass demnächst auch den Patienten mit HFpEF eine wirksame Behandlungsmöglichkeit durch SGLT2-Inhibitoren angeboten werden kann. Aus diesem Grund werden die Daten der DELIVER-Studie zu Dapagliflozin und der EMPEROR-preserved-Studie zu Empagliflozin mit großer Spannung erwartet [28–30].

## Einfluss der Gliflozine auf die Progression der Niereninsuffizienz

### Wichtige Studien

In den Studien CANVAS, EMPA-REG-OUTCOME und DECLARE-TIMI zeigte sich bereits eine Nephroprotektion durch die Behandlung mit Gliflozinen. Es handelt sich jedoch bei den renalen Endpunkten in diesen Studien nur um sekundären Endpunkte.

Die CREDENCE-Studie fokussierte im Jahr 2019 als Erste hauptsächlich auf den nephroprotektiven Effekt von Canagliflozin bei chronisch nierenkranken Patienten mit Diabetes mellitus und Albuminurie [31]. Canagliflozin in Kombination mit einem ACE-Hemmer reduzierte bei Patienten mit Typ-2-Diabetes und relevanter Proteinurie im Vergleich zur Behandlung mit einem Placebo das relative Risiko für renale Komplikationen um 30 % (HR 0,70; 95 %-KI 0,59–0,82, *p* < 0,0001). Dieser Effekt trat unabhängig vom Ausmaß der Senkung der Blutzuckerkonzentration, des Blutdrucks und des Körpergewichts auf. Die MACE-Rate wurde in der CREDENCE-Studie ebenfalls durch die Gliflozinbehandlung reduziert (HR 0,80; 95 % KI 0,67–0,95, *p* < 0,01).

Die DAPA-CKD-Studie wurde aufgrund der hohen günstigen Wirkung von Dapagliflozin vorzeitig gestoppt

Um den Effekt der SGLT2-Hemmer auf die Progression der Niereninsuffizienz weiterzuuntersuchen, wurde die Studie DAPA-CKD durchführt. Diese analysierte Die Wirkung von Dapagliflozin auf die Nierenfunktion und das Auftreten von kardiovaskulären Ereignissen bei nierenkranken Patienten [32]. Auch nicht an Diabetes erkrankte Patienten wurden in diese Studie eingeschlossen. Hierdurch unterscheidet sich DAPA-CKD von CREDENCE, da in die Letztere nur Patienten mit Typ-2-Diabetes aufgenommen wurden. Aufgrund der günstigen Wirkung von Dapagliflozin in einem unerwartet hohen Ausmaß wurde die Studie DAPA-CKD vorzeitig gestoppt [32]. Bis zu diesem Zeitpunkt waren in dieser Doppelblindstudie 4031 Patienten entweder mit Dapagliflozin, 10 mg/Tag, oder Placebo behandelt worden. Durch die Gabe von Dapagliflozin wurde der primäre Kombinationsendpunkt, bestehend aus einem 50 %igen Abfall der Nierenfunktion, dem Erreichen einer terminalen Niereninsuffizienz oder einem renalen oder kardiovaskulär-bedingten Todesereignis, von signifikant weniger Patienten in der Dapagliflozinguppe als in der Placebogruppe erreicht (HR 0,61; 95 %-KI 0,51–0,72; *p* < 0,001); dies entspricht einer relativen Risikoreduktion von 39 %. Das kombinierte Risiko für einen Tod aus kardiovaskulärer Ursache oder einer Krankenhauseinweisung wegen Herzinsuffizienz sank ebenfalls signifikant um 29 % ab (HR 0,71; 95 %-KI 0,55–0,92; *p* < 0,001).

### Fazit – zunehmende Evidenz für die nephroprotektive Wirksamkeit der Gliflozine auch bei Nicht-Diabetes-Patienten

Die in den 3 großen kardiovaskulären Endpunktstudien dokumentierte nephroprotektive Wirksamkeit der Gliflozine bei Menschen mit Typ-2-Diabetes führte dazu, dass große randomisierte Studien wie CREDENCE und DAPA-CKD durchgeführt wurden, um primär den renalen Effekt der verschiedenen SGLT2-Inhibitoren zu untersuchen. Ausstehend sind die Ergebnisse der Studie EMPA-KIDNEY [33]. In dieser multizentrischen randomisierten doppelblinden Studie werden derzeit ca. 6000 Patienten mit manifester chronischer Nierenerkrankung mit und ohne Diabetes untersucht. Vergleichbar mit der Studie DAPA-CKD wird hier erfasst, inwiefern Nicht-Diabetes-Patienten in gleicher Weise wie Diabetespatienten von einer nephroprotektiven Therapie mit Empagliflozin profitieren. Letztendlich trägt diese Studie dazu bei, die Wirkung von Gliflozinen auf das Fortschreiten der Niereninsuffizienz generell besser einschätzen zu können.
